# A Reassessment of Weaning Parameters in Patients With Spontaneous Intracerebral Hemorrhage

**DOI:** 10.7759/cureus.12539

**Published:** 2021-01-06

**Authors:** Paras Savla, Harjyot Toor, Stacey Podkovik, Joseph Mak, Sarala Kal, Chantal Soliman, Andrew Ku, Gohar Majeed, Dan E Miulli

**Affiliations:** 1 Neurosurgery, Riverside University Health System Medical Center, Moreno Valley, USA; 2 Internal Medicine, University of California Riverside School of Medicine, Riverside, USA; 3 Neurosurgery, St. George's University School of Medicine, St. George, GRD; 4 Neurosurgery, California University of Science and Medicine, Colton, USA; 5 Neurosurgery, Arrowhead Regional Medical Center, Colton, USA

**Keywords:** spontaneous breathing trial, intracerebral hemorrhage, rapid shallow breathing index, extubation, ich, rsbi, nif, tracheostomy, extubation failure, negative inspiratory force

## Abstract

Background and purpose

Patients with spontaneous intracerebral haemorrhage have significant morbidity and mortality. One aspect of their care is the need for mechanical ventilation. Extubating a patient safely and efficiently is important in advancing their care; however, traditional extubation criteria using the rapid shallow breathing index and negative inspiratory force do not predict success in these patients as well as they do in other intubated patients. This study aimed to evaluate these criteria in patients with spontaneous intracerebral haemorrhage to improve the extubation success rate.

Methods

We conducted a retrospective chart review of patients with spontaneous intracerebral haemorrhage (sICH) who underwent spontaneous breathing trials from 2018 to 2020. Twenty-nine patients met the inclusion criteria, and of these 29, 20 had a trial of extubation. Rapid shallow breathing index (RSBI), negative inspiratory force (NIF), and cuff leak were recorded to analyze breathing parameters at the time of extubation. Patients who required reintubation were noted.

Results

All trials of extubation required a cuff leak. Using RSBI, patients with values <105 or <85, as the only other extubation criteria, were associated with a 70.6% and 71.4% success rate, respectively. With RSBI <105 and NIF <-25 cm water, the success rate was 88.9%. Any patient with a cuff leak that had a NIF <-30 had a success rate of 100%, regardless of RSBI.

Conclusion

The RSBI was not a reliable isolated measure to predict 100% extubation success. Using a NIF <-30 predicts a 100% extubation success rate if a cuff leak is present. This demonstrates that the NIF may be a more useful metric in sICH patients, as it accounts for patient participation and innate ability to draw a breath spontaneously. Future studies are warranted to evaluate further and optimize the extubation criteria in these patients.

## Introduction

The outcome of spontaneous intracerebral haemorrhage (sICH) patients remains grim, especially in patients requiring mechanical ventilation. Families of sICH patients will often ask physicians to predict survival, independence, and need for prolonged ventilatory support. Although several prognostic models and scores were designed to predict favourable vs unfavourable outcome or death following sICH, none were outlined to predict the need for prolonged ventilation reliably and ultimately tracheostomy [1,2]. 

Many sICH patients with poor mental status are at risk of airway compromise and require intubation and mechanical ventilation (MV) [3]. Some will ultimately get extubated. The rest will require a tracheostomy for long-term airway support. In general, tracheostomy is usually indicated after 14-21 days of MV not only to prevent complications of prolonged intubation such as vocal cord injury, tracheomalacia, and ventilator-associated pneumonia but also to facilitate discharge of the patient from the intensive care unit (ICU) [4]. Extubation delay is associated with increased morbidity, mortality, length of ICU and hospital stay, and increased hospital charges [5]. However, failed extubation and the need for reintubation also serves as a major cause of morbidity and mortality in these patients [6]. 

Traditionally, two parameters in addition to cuff leak used in determining extubation trials are the RSBI and NIF. The RSBI is the ratio of respiratory frequency (f) to tidal volume (VT). The NIF relies on a patient’s ability to generate spontaneous breathing which occurs maximally by following commands. These values have been studied, and cutoffs have been generated to reach a satisfactory successful extubation rate [7,8]. However, these respiratory weaning parameters are not always reliable in brain injury patients, including those with sICH due to the inability to achieve maximal spontaneous inspiration [8,9]. Although several predisposing factors, such as the presence of obstructive lung disease, hematoma volume, poor Glasgow Coma Scale (GCS), basal ganglia haemorrhage, presence of hydrocephalus and loss of brainstem reflexes, suggest a greater likelihood of failed extubation, intensivists continue to use cuff leak, RSBI primarily, and NIF to make decisions regarding extubation trials [10,11].

Our study seeks to determine the optimal cutoffs of RSBI and NIF, in addition to cuff leak, for successful extubation in patients with sICH. We evaluate patients with sICH and their breathing parameters with reintubation and tracheostomy rates.

## Materials and methods

We conducted the study at a single institution using a retrospective chart review. Patients were selected from 2018 to 2020. The inclusion criteria were patients who were intubated, an age >18 years, the presence of sICH on computerized tomographic (CT) scans, who had undergone spontaneous breathing trials with recorded breathing parameters. The exclusion criteria were a diagnosis of traumatic brain injury, vascular malformations on cerebral imaging, patients who had not undergone brain CT scans without contrast on admission, and patients whose families had only elected for comfort measures. The Institutional Review Board at the institution approved this study.

The initial patient data were recorded from the time of admission, including the Glasgow Coma Scale (GCS), intracerebral haemorrhage (ICH) score, and the National Institutes of Health (NIH) Stroke Scale. The cerebral CT scan evidence of intracerebral haemorrhages was characterized by location, laterality, volume, intraventricular haemorrhage, amount of midline shift, and hydrocephalus presence. O2 saturation, RSBI, NIF, partial pressure of oxygen (pO_2)_, partial pressure of carbon dioxide (pCO_2)_, bicarbonate (HCO_3)_, and presence of cuff leak were recorded on each day of the spontaneous breathing trial. Patients were considered for extubation according to the criteria of RSBI <105, NIF <-25 cm H2O, and presence of cuff leak, along with a clinical judgment. Patient charts were then evaluated to determine which patients required reintubation and/or tracheostomy.

## Results

Twenty-nine patients met the inclusion criteria and were evaluated in the study. The characteristics of their intracerebral haemorrhages are outlined in table 1. As seen in table 2, of these 29 patients, 20 were extubated based on spontaneous breathing trial parameters and clinical judgment. The typical framework for parameters at our institution is the published standard of RSBI <105 and NIF <-25 cm H_2_O along with the presence of a cuff leak [7]; however, patients not meeting these requirements could undergo extubation if clinically indicated. Table 3 outlines the standard criteria’ success rates at our hospital, along with various other cutoffs for both RSBI and NIF. As noted in the table, cuff leak and NIF <-30 cm H_2_O, independent of RSBI, led to 100% successful extubation rate.

**Table 1:** Characteristics of intracerebral haemorrhages. ICH = intracerebral hemorrhage; n = number; IVH = intraventricular hemorrhage; ml = milliliters; mm = millimeters

ICH Parameters	
ICH Score	Patients (n=29)
1	5
2	14
3	7
4	3
5	0
Laterality	
Left	20
Right	8
Midline	1
Location	
Lobar	5
Basal ganglia/thalamus	23
Brainstem	1
IVH	
Yes	20
No	9
Hydrocephalus	
Yes	9
No	20
Average volume	20.5 ml
Average midline shift	3.5 mm

**Table 2:** Ventilation status of patient population.

Ventilation Status	Patients (n=29)
Extubated	20
Successful	14
Reintubated	6
Tracheostomy	15

**Table 3:** Extubation parameters along with success rate expressed in percentage. RSBI = rapid shallow breathing index; NIF = negative inspiratory force

Parameter(s)	Total Patients	Successful Extubations	Percent success (%)
RSBI <105 and NIF	9	8	88.9
RSBI <85 and NIF	7	7	100.0
RSBI <105	17	12	70.6
RSBI <85	14	10	71.4
NIF	11	10	90.9
NIF	9	9	100.0

Patients who failed extubation and ultimately underwent tracheostomy placement had various non-optimal spontaneous breathing parameters and continued to have non-optimal RSBI and NIF. Four patients met the criteria for extubation per cuff leak, RSBI and NIF, but ultimately were not extubated and underwent tracheostomy placement due to clinical judgment.

The positive predictive value for using NIF <-30 cm H_2_0 is 100%. The negative predictive value of using the latter is 50%.

## Discussion

The normal weaning criteria, RSBI <105 and NIF<-25 may not always apply to patients with intracerebral haemorrhages or other brain injuries [8,11,12]. Although a typical success rate of approximately 80% has been determined for extubation in patients who required prolonged mechanical ventilation [13], our patients were only successfully extubated 70% of the time. This further supports the notion that patients with a neurological injury who have an altered mental status, do not necessarily follow the normal paradigms seen in other ventilated patients. Additional care must be taken to prevent adverse outcomes from both too aggressive and not aggressive enough extubation. Patients risk developing tracheal and esophagal injuries from reintubation. Extended intubation can lead to pneumonia, more prolonged ICU stays, and the development of a poorer prognosis [5,14].

The RSBI has been traditionally verified to have a positive predictive value (PPV) and negative predictive value (NPV) of 79% and 95%, respectively, at a cutoff value of 105 when evaluating patients for extubation [7]. Prior studies have noted that neurosurgical patients are typically intubated for airway protection and not lung pathology and therefore, the RSBI is not necessarily a good predictor of extubation success. In the same study, only one patient that required reintubation had an RSBI >105, which would have predicted extubation success [15]. Due to the NIF being a measure of the inspiratory muscles, this measure may be the strongest indicator of extubation success [7,16]. The NIF considers a patient’s innate ability to generate inspiratory force to create a breath along with their ability to have cognitive input to maximize their effort. As such, the NIF inherently measures a patient’s ability to protect the airway than the RSBI better. As evidenced by our study, we can reach 100% success rate by altering the NIF goal independent of the RSBI criteria. Using NIF <-30 cm H2O as criteria, we have a positive predictive value of 100% and a negative predictive value of 50%, ensuring success. Even though the negative predictive value is low, patients do not suffer additional trauma and reintubation complications, which is more burdensome than temporary tracheostomy [6]. This further supports the notion that the NIF, in addition to cuff leak, maybe the most critical metric in determining successful extubation in these patients.

In patients that ultimately had a tracheostomy, the spontaneous breathing parameters fit within the newly proposed criteria, but other clinical factors determined the patients to not be candidates for extubation. These factors were the “standard” concerns for neurologically suppressed patients and included poor mentation as determined by the respiratory therapist’s judgment. One notable incident leading to reintubation in the presence of a new or worsening bleed, causing a deterioration in neurological status. These patients may again become unable to protect their airway and require reintubation; the RSBI and NIF cannot account for the risk of this occurring. This highlights the importance of clinical judgment when deciding to extubate. Clinical judgment typically supersedes numerical evaluation through spontaneous breathing trials and can lead to patients not being extubated at a high enough rate [12]. It is essential to note the subjectivity in clinical judgment, and having criteria with a high predictive value can help mitigate the concerns of operator differences.

Our study is not without its limitations. The patient population can be limited due to the nature in which patients arrive at the county hospital. Many patients have private insurance, requiring transfer to in-network facilities once stabilized, and many patients were transferred before extubation. A larger number of patients could provide further support for our conclusions. Additionally, many patients did not have reliable past medical histories and prior lung pathologies, which could have contributed to extubation success and failure due to an overabundance of caution about clinical judgment [13]. Analyzing these variables and others such as the route of intubation and degree of sedation in the failed extubations would help evaluate more possible causes instead of only assessing spontaneous breathing trial parameters [14].

## Conclusions

Extubation success for all critical care patients is of vital importance. The traditional weaning parameters of RSBI and NIF have been valuable in predicting this success; however, neurosurgical patients do not necessarily follow the same patterns of extubation criteria. We have demonstrated that RSBI may not be as vital in decision making as the use of NIF<-30 cm H_2_O. A stricter goal of a NIF<-30 cm H_2_O leads to a far higher success rate for extubations. Further prospective studies are needed to continue to test the weaning criteria in extubating neurosurgical patients with sICH.
